# Real-time kinetics of electrogenic Na^+^ transport by rhodopsin from the marine flavobacterium *Dokdonia sp.* PRO95

**DOI:** 10.1038/srep21397

**Published:** 2016-02-11

**Authors:** Alexander V. Bogachev, Yulia V. Bertsova, Marina L. Verkhovskaya, Mahir D. Mamedov, Vladimir P. Skulachev

**Affiliations:** 1 Belozersky Institute of Physico-Chemical Biology, Lomonosov Moscow State University, Moscow 119234, Russia; 2 Institute of Biotechnology, PO Box 65 (Viikinkaari 1) FIN-00014, University of Helsinki, Finland

## Abstract

Discovery of the light-driven sodium-motive pump Na^+^-rhodopsin (NaR) has initiated studies of the molecular mechanism of this novel membrane-linked energy transducer. In this paper, we investigated the photocycle of NaR from the marine flavobacterium *Dokdonia sp.* PRO95 and identified electrogenic and Na^+^-dependent steps of this cycle. We found that the NaR photocycle is composed of at least four steps: *NaR*_519_ + *hv* → *K*_585_ → (*L*_450_↔*M*_495_) → *O*_585_ → *NaR*_519_. The third step is the only step that depends on the Na^+^ concentration inside right-side-out NaR-containing proteoliposomes, indicating that this step is coupled with Na^+^ binding to NaR. For steps 2, 3, and 4, the values of the rate constants are 4×10^4^ s^–1^, 4.7 × 10^3^ M^–1^ s^–1^, and 150 s^–1^, respectively. These steps contributed 15, 15, and 70% of the total membrane electric potential (Δψ ~ 200 mV) generated by a single turnover of NaR incorporated into liposomes and attached to phospholipid-impregnated collodion film. On the basis of these observations, a mechanism of light-driven Na^+^ pumping by NaR is suggested.

Transformation of solar energy plays a key role in the functioning of the Earth’s biosphere. So far, two fundamentally different ways used by living organisms to store energy of light quanta have been discovered: (i) chlorophyll-containing photosynthetic complexes, and (ii) retinal-containing proteins (bacteriorhodopsin and halorhodopsin). The latter mechanism is characterized by lower efficiency of energy conversion compared to chlorophyll-containing photosystems. However, the organization of retinal-containing proteins is much simpler compared to chlorophyll-containing reaction centers. Bacteriorhodopsin consists of only one, rather small subunit, with a single prosthetic group (retinal). These properties probably allowed the protein to survive in the course of biological evolution up to the present day and to play a noticeable (though quantitatively rather small) role in solar energy conversion in the biosphere.

The retinal moiety is covalently bound in the bacteriorhodopsin molecule by means of an aldimine bond with the ε-amino group of a lysine residue. Photon absorption causes retinal isomerization (all-*trans* → 13*-cis*). This process is accompanied by a strong decrease in pK_а_ of the Schiff base imine nitrogen, which is protonated in the dark form of bacteriorhodopsin. As a result, the Schiff base is deprotonated, and the proton is released into the aqueous phase outside the bacterial cell. Then the рK_а_ of the Schiff base increases to the initial value, which results in its protonation by an intracellular H^+^. In the last stage, retinal returns to its original conformation (13*-cis* → all*-trans* transition). In this way, the light-induced cyclic transformation of bacteriorhodopsin is coupled to uphill Н^+^ transport across the cytoplasmic membrane^1^,^2^.

Bacteriorhodopsin is a single polypeptide; its mass is ~26 kDa, which is considerably lower than that of all other known Δ
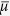
_H_^+^-generators^3^. Bacteriorhodopsin has specific properties that facilitate its study. Among them there are strong light absorption bands differing in the dark bacteriorhodopsin form and intermediates of its catalytic cycle (photocycle) in the visible spectral range; possibility to induce the transmembrane H^+^ transfer by a single laser flash; ability to form both two-dimensional crystals in purple membranes and three-dimensional crystals in an artificial cubic phase of lipids; exceptional stability of bacteriorhodopsin over a very wide range of organic solvent concentrations, pH values, pressures, temperatures, etc. As a result, bacteriorhodopsin is the most thoroughly studied proton pump. Complete identification of the mechanism of its functioning should facilitate understanding of the mechanisms of other proton-translocating proteins.

Previously, various retinal-containing proteins were found to function as light-dependent pumps of H^+^ or Cl^−^ and as light receptors^2^,^4^. Recently, it was found that one of the rhodopsins of the marine flavobacterium *Krokinobacter eikastus* NBRC 100814T is capable of light-dependent transport of Na^+^ from the cytoplasm to the external medium^5^. In the absence of Na^+^, this protein transfers H^+^ in the same direction, albeit at a much lower rate^5^. In terms of its ability to transport both sodium and hydrogen ions, Na^+^-rhodopsin (NaR) is reminiscent of some Na^+^-translocating ATPases and pyrophosphatases^6^. Later, NaR was also found in some other marine flavobacteria: *Nonlabens marinus* S1-08T^7^, *Gillisia limnaea* R-8282T^8^, *Dokdonia sp.* PRO95^9^, and *Nonlabens dokdonensis* DSW-6 10. Analysis of amino acid sequences of Na^+^-rhodopsins shows that they form a separate phylogenetic group among retinal-binding proteins^10^. Recently, the three-dimensional structure of NaR from *K. eikastus* NBRC 100814T was determined by X-ray structural analysis^11^,^12^. This approach revealed a number of structural properties specific for NaR that are absent from other retinal-dependent ion pumps^11^,^12^.

Studies of Na^+^-translocating enzymes have a number of advantages over the H^+^-pumps. When studying Na^+^-pumps, we can freely manipulate the pumped ion concentration without significant effect on the stability of the protein, which is impossible in the case of H^+^-translocating enzymes. In particular, we can study the catalytic cycle of the sodium-dependent enzyme when limiting its rate by low Na^+^ concentration and identify the stages that are specifically accelerated by Na^+^. Therefore, studying Na^+^-translocating rhodopsins should lead to significant progress in understanding of the functional mechanism of retinal-containing energy-transforming enzymes.

Direct electrometry is a convenient method for detecting transmembrane transfer of charged species by proteins operating as generators of transmembrane electric potential (Δψ). This approach has been successfully used in studies of bacterial photosynthetic reaction centers and cytochrome *bc*_1_-complex^13^,^14^,^15^, pigment–protein complexes of photosystems II and I^16^,^17^, terminal oxidases^18^,^19^,^20^,^21^, bacteriorhodopsin^22^,^23^, and halorhodopsin^24^. In the present paper, we used this method to study the catalytic cycle of the recently described NaR.

## Results

### Generation of transmembrane electric potential difference in individual steps of the NaR photocycle

**Figure 1:**
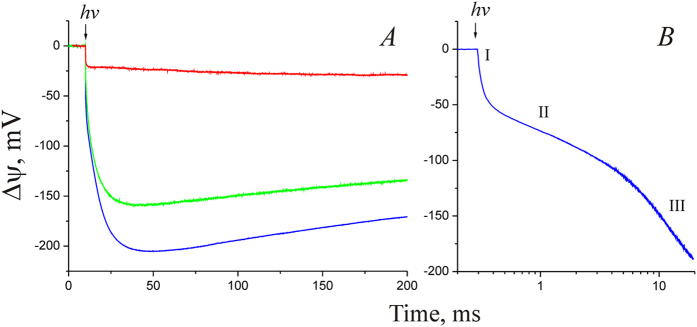
Direct measurement of Δψ generation by NaR-proteoliposomes adsorbed on a phospholipid-impregnated collodion film. (**A**) The medium contained 100 mM KCl (red curve), 100 mM NaCl (blue curve), or 100 mM LiCl (green curve). (**B**) Photoelectrogenic response of NaR in medium containing 100 mM NaCl shown on a semilogarithmic scale. Roman numbers indicate three steps of Δψ generation.

The NaR gene from *Dokdonia sp.* PRO95 was heterologously expressed in *Escherichia coli* cells; then NaR was isolated by affinity chromatography and incorporated into liposomes. Finally, the NaR-proteoliposomes were adhered to a phospholipid-impregnated collodion film. Figure 1A (blue curve) shows that illumination of the film-adhered NaR-proteoliposomes with a single laser flash (in the presence of 100 mM NaCl inside and outside of the proteoliposomes) resulted in the generation of a transmembrane electric potential difference (Δψ) of ~220 mV. The sign of Δψ indicated that positive charge was transported from the interior of the proteoliposomes to the outer medium, i.e. the NaR orientation in proteoliposomes is right-side-out. Analysis of the kinetics of the photoelectric response revealed (i) three successive steps of Δψ generation (Fig. 1B; time constants, 25 μs, ~1.5 ms, and ~7 ms) and (ii) the Δψ decay; the time constant of the decay varied in different preparations from fractions of a second to several seconds. The contribution of the three detected phases of Δψ generation into the total Δψ was about 15%, 15%, and 70%, respectively.

The laser flash illumination of proteoliposomes containing 100 mM KCl both inside and outside (background Na^+^ concentration in the medium was about 30 μM) resulted in a photoresponse of quite different shape and very much lower amplitude than in 100 mM NaCl (Fig. 1A, red line). Kinetic analysis of Δψ generation in 100 mM KCl revealed only one definite phase with measurable τ (25 μs). Subsequent phase(s) were so slow that they merged with the Δψ decay, a fact that made it impossible the reliable determination of their kinetic characteristics. Thus, these data confirmed our earlier conclusion about the rhodopsin from *Dokdonia sp.* PRO95 being a primary photoelectrogenic Na^+^-pump^9^.

Previously, it was shown that NaR from *K. eikastus* NBRC 100814T could pump not only Na^+^, but also lithium ions^5^. To test this observation on NaR from *Dokdonia sp.* PRO95, we studied the kinetics of light-induced Δψ generation in the presence of 100 mM LiCl (inside and outside of the proteoliposomes). As shown in Fig. 1A (green curve), proteoliposomes illumination with a laser flash in a Li^+^-containing medium resulted in Δψ generation with kinetics similar to that of Na^+^-containing medium, which indicates the ability of NaR to pump not only sodium, but also lithium ions.

Thus, we identified three kinetic steps of transmembrane charge transfer coupled to the light-induced turnover of NaR in Na^+^- or Li^+^-containing media. Addition of NaCl to proteoliposomes prepared in KCl-containing medium did not lead to an increase in the photoresponse amplitude or acceleration of any phase of Δψ generation (data not shown). This observation is consistent with the right-side-out orientation of NaR in the proteoliposomes (see above), i.e. the Na^+^-linked Δψ generation requires Na^+^ binding by NaR from the interior of vesicles impermeable for this ion. In our experiments, the effect of externally added NaCl on the kinetics of Δψ generation could be achieved in the presence of the Na^+^/H^+^-exchanger monensin (data not shown). However, due to the large volume of the hydrophobic phase in our experimental system (phospholipid-impregnated collodion film), rather high concentration of this exchanger (~5 μM) was required to achieve the effect. However, high monensin concentration is known to cause a reduction of Δψ^25^. Accordingly, addition of 5 μM monensin caused not only the acceleration of Δψ generation, but also stimulation of its decay, which complicated the use of this exchanger in our experiments. Therefore, to determine the Na^+^ dependence of Δψ generation, we used a series of proteoliposome preparations prepared at different Na^+^ concentrations.

**Figure 2:**
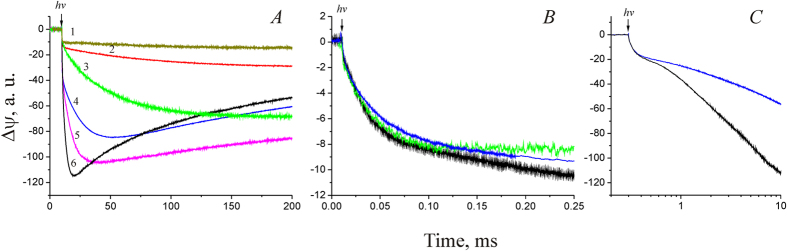
Generation of Δψ by NaR at different NaCl concentrations that were equal outside and inside proteoliposomes. Experiments were performed in medium containing 20 mM HEPES-Tris, pH 7.5, 100 mM KCl, and varying NaCl concentrations. (**A**) Kinetics of Δψ generation in the time scale 0–200 ms. Curves 1–6 correspond to NaCl concentrations of 0.03, 1, 4, 16, 64, and 200 mM, respectively. (**B**) Kinetics of Δψ generation in the time scale 0–250 μs in the presence of 100 mM KCl (green curve) or NaCl (black curve) in H_2_O or in the presence of 100 mM NaCl in D_2_O (blue curve). Amplitudes of the responses were normalized to the amplitudes of their 25 μs phase. (**C**) Kinetics of Δψ generation by NaR at 200 mM NaCl concentration in medium containing H_2_O (black curve) or D_2_O (blue curve).

The kinetics of Δψ generation by NaR-containing proteoliposomes at various Na^+^ concentrations are shown in Fig. 2, and analysis of the data is summarized in Table 1. It is important to note that these results were obtained on different proteoliposome preparations. Therefore, in these experiments we compared the kinetics of electric potential generation and the relative contribution of the different phases in Δψ generation, but not the absolute values of the amplitudes of the responses. As shown in Fig. 2B, the kinetics of the fastest phase of Δψ generation did not depend on Na^+^ concentration; its time constant was ~25 μs in all cases. The slowest, millisecond phase of Δψ generation was significantly accelerated over the entire range of Na^+^ concentrations (increasing from 30 μM to 200 mM; Fig. 2A). Increasing [Na^+^] from 1 to 200 mM reduced the time constant of this phase 35-fold (from 160 to 4.5 ms). We could not identify the Na^+^-dependence of the intermediate (sub-millisecond) phase. This phase was detected only at NaCl concentration from 60 to 200 mM. At lower Na^+^ concentrations, this phase could not be detected, presumably because its deceleration caused its merging with the subsequent millisecond phase of Δψ generation. It must be noted that while varying NaCl concentrations in these experiments, we did not keep constant ionic strength. Thus, the influence of varying ionic strength on the NaR photoresponse cannot be excluded. However, all measurements were done with a high background KCl concentration (100 mM), which makes this effect likely small or negligible.

The kinetics of Δψ generation by NaR reconstituted into liposomes was also measured in a D_2_O containing medium (Fig. 2B,C). Analysis of these data (Table 1) revealed that the H_2_O/D_2_O substitution resulted in about 1.4- and 2-fold deceleration of the first and third phases, respectively. In contrast, the H_2_O/D_2_O substitution had no effect on the rate of the intermediate phase (phase II).

### Optical studies of the NaR photocycle as a function of the Na^+^ concentration

**Figure 3:**
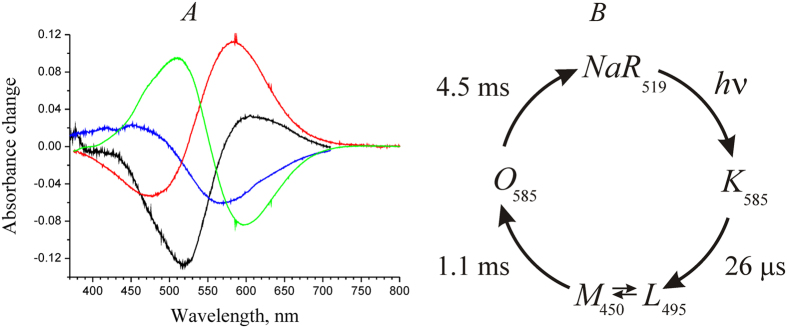
Photocycle of NaR in proteoliposomes. (**A**) Differential optical spectra of light-induced transitions in NaR. Black curve, *NaR* 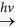 *K*; blue curve, *K* → (*L*↔*M*); red curve, (*L*↔*M*) → *O*; green curve, *O* → *NaR*. (**B**) Scheme of NaR photocycle in proteoliposomes at [Na^+^] = 200 mM.

Initial experiments on the photocycle were carried out on NaR-containing proteoliposomes with 200 mM NaCl concentration both inside and outside the proteoliposomes. We observed four light-induced transitions accompanied by changes in light absorption by NaR. The optical spectra (Fig. 3A) and characteristic times (Fig. 3B) of these transitions were identified. The following observations were described:

- Light absorption by the dark NaR form produced a time-unresolved shift of the retinal absorption maximum to the red region (from 519 to ~585 nm, formation of intermediate *K*, black curve).
- Then, the absorption band shifted to the blue region, an event accompanied by the formation of two new peaks, i.e. a minor one at ~450 and a major one at ~495 nm (time constant, 26 μs, blue curve). Consistent with the earlier reported data^5^, this intermediate can be described as an equilibrium between forms *L* and *M* (*L*↔*M*).
- Later, the intermediate *O* (absorption maximum, ~585 nm) was formed with time constant of 1.1 ms (red curve).
- In the last stage of the photocycle, intermediate *O* is converted to the initial dark form of NaR (time constant, 4.5 ms, green curve).

These data (both spectral properties of the intermediates and characteristic times of their formation) are in good agreement with the previously described photocycle of NaR from *K. eikastus* NBRC 100814 T^5^. It is also important to note the good match between the kinetics of intermediate formation in the NaR photocycle and characteristic times of electric potential generation (see above, Table 1).

Since changing of Na^+^ concentration inside of proteoliposomes faces certain difficulties (see above), further study of Na^+^-dependent kinetics of the formation of NaR photocycle intermediates was performed using NaR solubilized with a detergent. The photocycle of the solubilized NaR in 200 mM NaCl was similar to the above-described photocycle of the protein in proteoliposomes. The only observed difference was 1.5-fold deceleration of the last stage of the photocycle (*O* → *NaR* transition) in the solubilized form of NaR.

**Figure 4:**
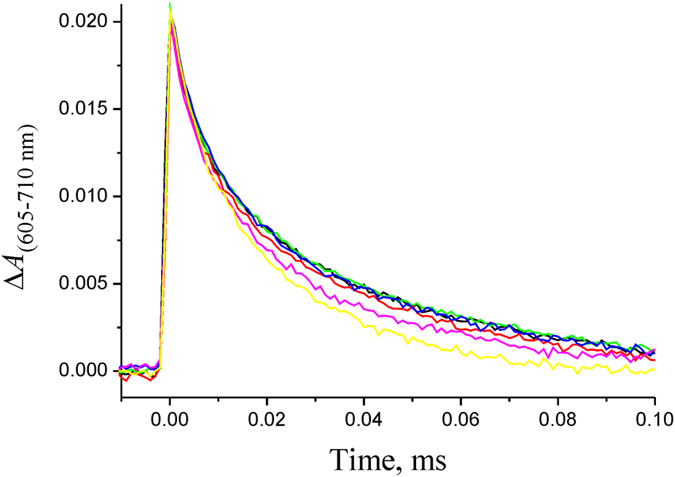
Kinetics of intermediate *K* decay in the NaR photocycle measured at λ = 605 nm at various NaCl concentrations. Experiments were performed in medium containing 20 mM HEPES-Tris, pH 7.5, 0.05% *n*-dodecyl β-d-maltoside, 100 mM KCl, and varying NaCl concentrations. The detergent-solubilized NaR was illuminated by the laser flash at zero time. Black, green, red, blue, purple, and yellow curves correspond to NaCl concentrations of 0.03, 1, 4, 16, 64, and 200 mM, respectively.

We studied the kinetics of the NaR photocycle at varying Na^+^ concentrations (30 μM–200 mM) in detergent solubilized NaR. Similarly to the results obtained on NaR from *G. limnaea* R-8282T^8^, decay of intermediate *K* (Fig. 4) was best described by the sum of two exponential processes with characteristic times of 7 and 40 μs. The optical spectra of these two processes were very similar (data not shown) and analogous to the spectrum of *K* → (*L* ↔ *M*) transition shown in Fig. 3A. Thus, it seems likely that the biphasic character of this process does not result from the presence of some additional intermediate in the NaR photocycle; it rather reflects heterogeneity of the studied preparation (various levels of protonation of a protein group at the given pH; different NaR oligomeric state, etc.). As a result, we later considered this transition as a monoexponential process with time constant of ~25 μs. As shown in Fig. 4, the rate of the *K* → (*L* ↔ *M*) transition did not depend on Na^+^ concentration. A minor apparent acceleration of this process at [Na^+^] > 60 mM was related not to decrease in the corresponding time constants, but rather to a minor change in the proportions of the 7- and 40-μs phases in the total *K* → (*L*↔*M*) transition. Thus, we conclude that the decay rate of intermediate *K* does not depend on Na^+^ concentration.

**Figure 5:**
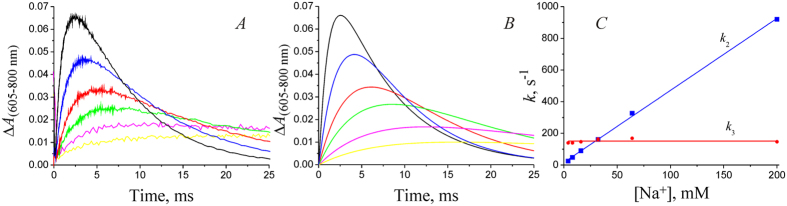
Kinetics of the formation and decay of intermediate *O* in the photocycle of detergent-solubilized NaR, measured at λ = 605 nm at different NaCl concentrations. (**A,B**) Experimental data and model curves obtained by fitting according to equation 1 (see text), respectively. Yellow, purple, green, red, blue, and black curves correspond to NaCl concentrations of 4, 8, 16, 32, 64, and 200 mM, respectively. Experimental conditions as in Fig. 4. (**C**) Dependence of kinetic constants of (*L*↔*M*) → *O* (*k*_2_) and *O* → *NaR* (*k*_3_) transitions on Na^+^ concentration.

**Figure 6:**
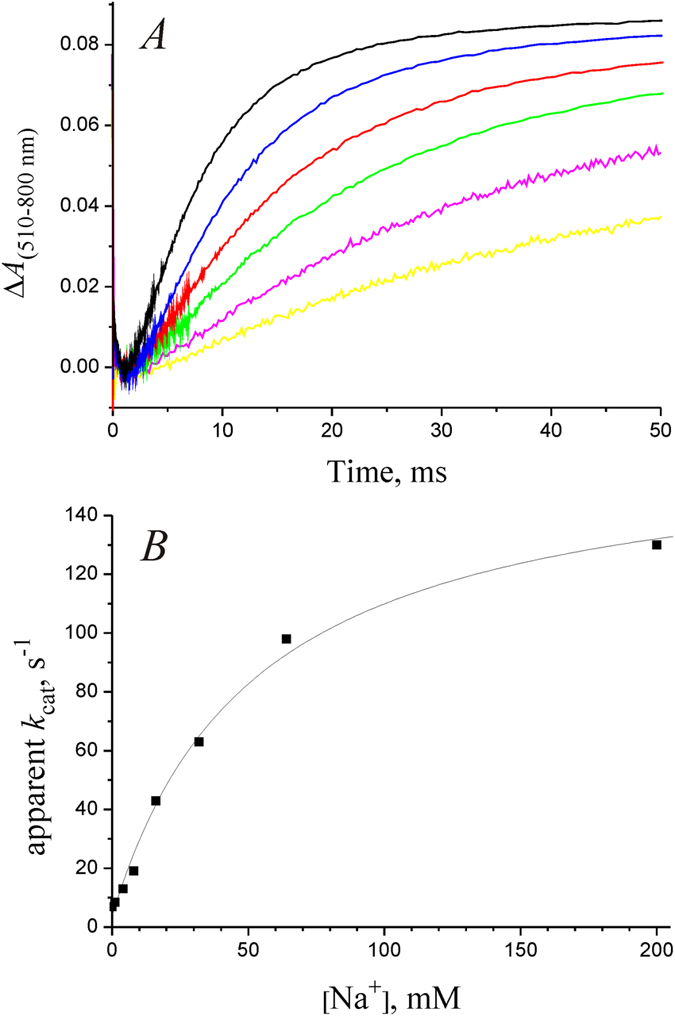
Kinetics of *O* → *NaR* transition in photocycle of detergent-solubilized NaR, measured at λ = 510 nm. (**A**) Experimental data obtained at various NaCl concentrations; yellow, purple, green, red, blue, and black curves correspond to NaCl concentrations of 4, 8, 16, 32, 64, and 200 mM, respectively. Experimental conditions as in Fig. 4. (**B**) Dependence of the NaR photocycle rate on Na^+^ concentration. Squares correspond to experimental data shown in (**A**), solid line shows fitting of the data using the Michaelis–Menten equation with 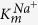 = 52 ± 8 mM.

The kinetics of the (*L*↔*M*) → *O* and *O* → *NaR* transitions at varying Na^+^ concentrations is presented in Figs 5A and 6A, respectively. In line with the previously described photocycle of NaR from *K. eikastus*^5^, both the formation of intermediate *O* and its decay were significantly accelerated at increased [Na^+^]. Acceleration of these processes resulted in about 20-fold increase in the total rate of the photocycle when Na^+^ concentration was increased from 30 μM to 200 mM (Fig. 6B). The resulting dependence was hyperbolic with 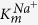 = 52 ± 8 mM.

The above-mentioned acceleration of the formation and decay of intermediate *O* can be explained in two different ways: (i) the rates of both of these transitions depend on [Na^+^]; (ii) only the rate of (*L*↔*M*) → *O* transition really depends on Na^+^ concentration, while the seeming dependence of the rate of *O* → *NaR* transition is because this photocycle transition occurs *after* the true Na^+^-dependent stage^5^. To answer this question, we performed fitting of the data on the kinetics of intermediate *O* formation and decay (Fig. 5A) using the approach described in the “Methods” section. According to equations (4) and (5) from that section, change in the optical density caused by the change in concentration of the third intermediate in the chain of sequential reactions is described by the following equation (1):


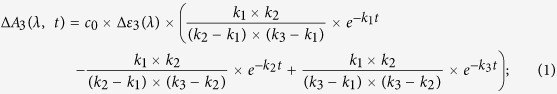


where *c*_0_ stands for the concentration of intermediate *K* immediately after the laser flash (at time zero), Δε_3_(λ) – extinction coefficients related to formation and decay of intermediate *O* at the selected wavelength, and *k*_1_, *k*_2_, and *k*_3_ are the rate constants of the *K* → (*L*↔*M*), (*L*↔*M*) → *O*, and *O* → *NaR* transitions, respectively. Taking into account *k*_1_ = 4×10^4^ s^–1^ (see above), we fitted the kinetics of intermediate *O* formation and decay according to equation (1) at 200 mM NaCl, i.e. for the conditions when *k*_2_ and *k*_3_ differ significantly from each other (Fig. 5A). This allowed us to reliably determine such parameter as the maximal possible amplitude of the optical density changes on the formation of the intermediate *O* [*c*_0_×Δε_3_(605 nm) = 0.095], which is common to all the curves in Fig. 5A (except varying Na^+^ concentration, these experiments were carried out under identical conditions with the same NaR concentration). Thereafter, all the dependences shown in Fig. 5A were fitted according to equation (1) at fixed parameters *k*_1_ = 4×10^4^ s^–1^ and *c*_0_ × Δε_3_ (605 nm) = 0.095, thereby determining the values of constants *k*_2_ and *k*_3_ at different Na^+^ concentrations. The resulting model curves of the changes in optical density during the formation and decay of intermediate *O* are shown in Fig. 5B. The dependence of the resulting *k*_2_ and *k*_3_ values on [Na^+^] is shown in Fig. 5C. As seen, the *k*_2_ value (rate constant of the (*L*↔*M*) → *O* transition) linearly depends on Na^+^ concentration in the entire range, while the *k*_3_ value (rate constant of the *O* → *NaR* transition) is Na^+^-independent. Thus, we conclude that the true rate of the *O* → *NaR* transition does not depend on Na^+^ concentration, and its apparent dependence is because this reaction follows the authentic Na^+^-dependent step in the photocycle. So, the data indicate that the (*L*↔*M*) → *O* transition is the true Na^+^-dependent step of the NaR photocycle, and it is this transition that is coupled to the Na^+^ binding by NaR for its further pumping. The data shown in Fig. 5C also provide basis for explanation of the determined 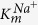 value of NaR operation (Fig. 6B). At Na^+^ concentration of about 50 mM, the rate of the (*L*↔*M*) → *O* transition (*k*_2_) becomes faster than the rate of the following Na^+^-independent *O* → *NaR* step (*k*_3_), and further increase in Na^+^ concentration does not result in significant acceleration of the NaR photocycle. It is noteworthy that in this case the 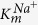 value cannot be used as a measure of NaR affinity to Na^+^ (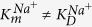).

It is important to note that the rate constant of the (*L*↔*M*) → *O* transition linearly depended on Na^+^ concentration in the measuring medium. The same linear dependence was recently described also for NaR from *G. limnaea* R-8282 T^8^. These data indicate that for all used [Na^+^], the rate of the (*L*↔*M*) → *O* step was limited by the rate of diffusion of the pumped Na^+^ to its binding site in NaR. Based on the slope of the dependence of *k*_2_ on [Na^+^], we determine the second-order rate constant 4.7 × 10^3^ M^–1^ s^–1^ for the bimolecular reaction 
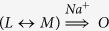
. Since the rate of this transition is limited by the Na^+^ diffusion to the site of its binding in NaR, we conclude that the kinetic constant of this ion binding 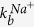
≈ 4.7 × 10^3^ M^–1^ s^–1^.

## Discussion

**Figure 7:**
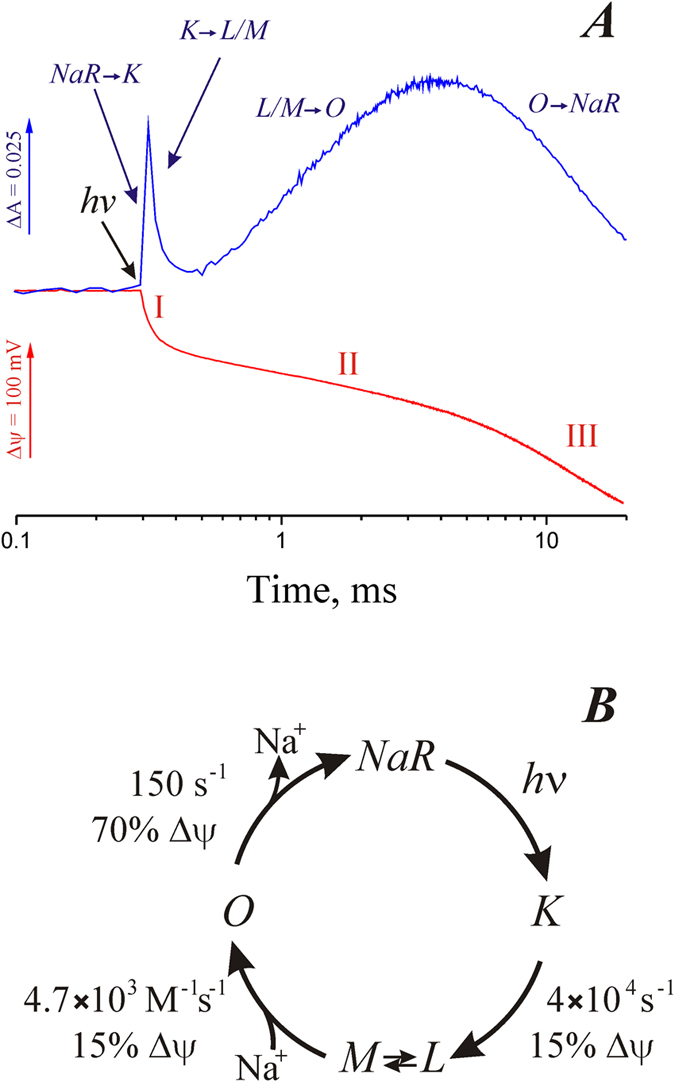
Kinetics of the NaR photocycle. (**A**) Kinetics of the NaR photocycle measured in proteoliposomes in medium containing 100 mM NaCl by absorbance changes at 605 nm (blue curve) or by Δψ generation (red curve). Roman numbers indicate three steps of Δψ generation. (**B**) Scheme of the NaR photocycle.

In this paper, we studied kinetics of the electrogenic response of the Na^+^-pumping rhodopsin from *Dokdonia sp.* PRO95 at different Na^+^ concentration. Using optical spectroscopy, we also determined the main intermediates of this cycle, kinetic constants of their formation and decay, as well as dependence of the rate of these processes on [Na^+^]. Due to the good match between the kinetics of intermediates formation in the NaR photocycle and electric potential generation (Fig. 7A), it can be concluded that the phases I, II, and III of Δψ formation correspond to the *K* → (*L*↔*M*), (*L*↔*M*) → *O*, and *O* → *NaR* transitions, respectively. Using the direct electrometry approach, we determined for the first time the contribution of the particular photocycle phases to the entire transmembrane charge transfer by this protein. The data are presented as a scheme of the NaR photocycle in Fig. 7B.

**Figure 8:**
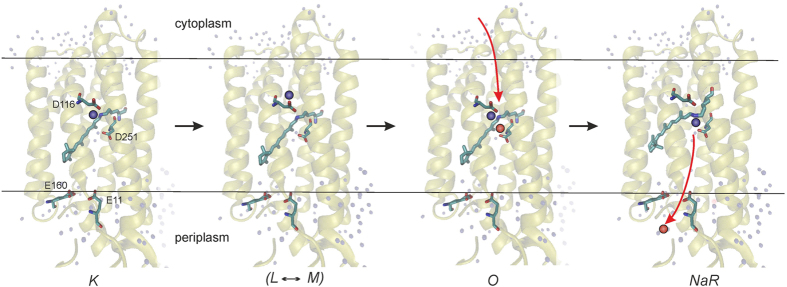
Scheme of transmembrane Na^+^ translocation by NaR. Protein structure is shown according to Gushchin *et al.*^11^. The Shiff base proton and pumped Na^+^ are indicated by blue and red spheres, respectively. Na^+^ movement is shown by red arrows.

Light absorption by the dark form of NaR leads to the *NaR* → *K* transition. This process was faster than the time resolution of our instruments and it was not accompanied by a measurable level of Δψ generation. Then intermediate *K* decays to the *L* and *M* forms, which are apparently in equilibrium with each other (*L*↔*M*). This transition was accompanied by a relatively small electrogenesis, which comprises about 15% of one charge transfer across the membrane. The characteristic time of this process was about 25 μs. It is important to note that the *K* → (*L*↔*M*) transition had identical kinetics in the absence or in the presence of Na^+^ when it was studied either by electrometry or by optical absorbance (Figs 2B and 4, respectively). Thus, this phase cannot be coupled to Na^+^ binding, and the Δψ generation during the *K* → (*L*↔*M*) transition should reflect either the release of the pre-bound Na^+^, or a light-induced H^+^ transfer in the hydrophobic part of NaR. The primary structure of NaR from *Dokdonia sp.* PRO95 is highly similar to the well-studied NaR of *K. eikastus* NBRC 100814T (98% identity). Previously, it was found that the dark form of NaR from *K. eikastus* contains a bound Na^+^ with K_D_ ≈ 11 mM^5^. However, we observed Δψ generation during the *K* → (*L*↔*M*) transition also in virtually sodium-free medium (the trace sodium concentration was about 30 μM), i.e. under conditions when [Na^+^] ≪ K_D_ (Fig. 2B). Moreover, the recently determined three-dimensional structure of *K. eikastus* NaR revealed that in the ground state this sodium ion is bound in the interface between NaR monomers in oligomers^11^. Thus, electrogenicity of the *K* → (*L*↔*M*) transition cannot be ascribed to Na^+^ transfer; it seems to be connected to H^+^ movement in the hydrophobic part of the protein. This conclusion is supported by 1.4-fold deceleration of the rate of Δψ formation during the *K* → (*L*↔*M*) transition in D_2_O (Table 1). On the basis of structural studies of NaR, it was previously suggested that the *K* → *M* transition is accompanied by H^+^ transfer from the Schiff base to the nearby residue D116^12^. However, because this H^+^ transfer proceeds almost parallel to the membrane plane, it can hardly itself explain the Δψ generation at this step (Fig. 8). Structural studies of NaR revealed that D116 is connected to D251 via a long chain of hydrogen bonds, and protonation of D116 results in the reorientation of this amino acid residue^12^. Thus, to account for the amplitude of Δψ generation coupled to the *K* → (*L*↔*M*) transition, we propose that the rearrangement of the hydrogen bonds between D116 and D251 in the *M* intermediate results in net charge transfer to the periplasmic surface of NaR. Also by analogy with the functioning of the H^+^-pumping bacteriorhodopsin, the *K* → (*L*↔*M*) transition can also be coupled with H^+^ release into periplasm from one of the glutamic acid residues located close to the surface (E11 or E160) (Fig. 8).

The next step of the NaR photocycle is the (*L*↔*M*) → *O* transition. It was significantly stimulated by Na^+^. This stimulation was caused by Na^+^ only inside right-side-out-oriented proteoliposomes, which corresponds to the cytoplasmic Na^+^ in the bacterial cell. Thus, the (*L*↔*M*) → *O* transition should be coupled to Na^+^ binding with NaR from the *cytoplasmic* side of the membrane. The rate constant of this transition linearly depended on Na^+^ concentration (Fig. 5C). The linear character of the dependence indicates that at all used Na^+^ concentrations, the rate of the (*L*↔*M*) → *O* stage is limited by the rate of Na^+^ diffusion to its binding site in NaR. Limitation of the (*L↔M*) → *O* transition by Na^+^ diffusion is also supported by absence of the D^+^/H^+^ isotope effect on the rate of Δψ formation during this transition (Table 1). Thus, dependence of the rate of the (*L↔M*) → *O* transition on Na^+^ concentration can be used to determine the kinetic binding constant of this ion as 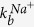
≈ 4.7×10^3^ M^–1^ s^–1^. Typically, the rate constant of Na^+^ binding with macromolecules is in the range 10^7^ – 10^9^ M^–1^ s^–1^ 26,27,28. Thus, Na^+^ diffuses to its final binding site in NaR at least four orders of magnitude slower than in most other sodium-binding proteins. Such hindered diffusion indicates that the site of Na^+^ binding in NaR is located at the bottom of a rather long and narrow cleft in the protein molecule. Recently reported movements of helices in the *K. eikastus* (*Dokdonia eikasta*) NaR resulted in E → C conformational change^29^ may be involved in formation of this cleft. Based on the spectral and structural data, it had been previously suggested that Na^+^-binding site could be located in the vicinity of the Schiff base^8^,^12^,^30^ (Fig. 8). This makes the path from the cytoplasmic membrane side to the binding site ~50–55% of the membrane thickness. As determined in the current work, the (*L*↔*M*) → *O* stage is accompanied by only a relatively small electrogenesis, about 15% of a single charge transfer across the membrane, which seems to contradict with localization of the Na^+^ binding site deeply embedded inside NaR. This contradiction might be partially explained by the back movement of the proton to the Schiff base, which is thought to occur at this stage^12^. But even in this case, electrogenesis associated with the Na^+^ movement in NaR should be ~30% of one charge transfer across the entire thickness of the membrane. However, this figure is still significantly lower than the length of about 50–55% of the membrane thickness. Thus, either (i) the Na^+^ binding site is located far from the Schiff base (somewhere halfway between the Schiff base and the cytoplasmic membrane surface), or (ii) Na^+^ binding takes place at the bottom of a deep and narrow, but *water-filled*, funnel characterized by a high value of dielectric constant. In the latter case, the funnel remains open during the entire (*L*↔*M*) → *O* transition.

The last event in the photocycle of the Na^+^-pumping rhodopsin is the *O* → *NaR* transition. This transition represents the main electrogenic stage of the NaR photocycle. Its contribution was about 70% of the total electrogenesis. The rate constant of this transition did not depend on Na^+^ concentration. Thus, the *O* → *NaR* transition is apparently accompanied by (i) closing of the above postulated cleft leading from the cytoplasmic side of the membrane to the Schiff base, and (ii) Na^+^ transfer from its binding site to the outer surface of the membrane (Fig. 8). High D^+^/H^+^ isotope effect on the rate of Δψ formation during the *O* → *NaR* transition (Table 1) indicated that this transition is limited by conformational changes in NaR.

Studies on the atomic structure of NaR^11^,^12^ and its molecular mechanism (this report), together with quite recent discovery of Na^+^-translocating cytochrome oxidase^31^ completed description of the main types ion pumps involved in the sodium cycle^32^, a mechanism of membrane bioenergetics alternative to the Mitchellian proton cycle^33^. The Na^+^ cycle not only extends the area of membrane bioenergetics to conditions where a proton cycle cannot be effective (high pH values or high membrane conductance to H^+^) but, after discovery of NaR^5^, might be considered as the primary mechanism of production of consumable energy in the biosphere^34^.

## Methods

### Purification of Na^+^-pumping rhodopsin

Heterologous expression of the gene of NaR from *Dokdonia sp.* PRO95 in *Escherichia coli* BL21 and isolation of the recombinant 6×His-tagged protein was performed as described previously^9^.

### Reconstitution of proteoliposomes with NaR

Proteoliposomes for Δψ generation experiments were prepared as follows. One milliliter of Buffer A [20 mM HEPES-Tris, pH 7.5, containing 100 mM KCl (NaCl or LiCl), 0.5% (w/v) Triton X-100, and 10 mg soybean phosphatidylcholine (Sigma, type IV-S, 40% (w/w) phosphatidylcholine content)] was sonicated until clarity. NaR was then added to this suspension at the lipid/protein ratio of 100:1 (w/w) and incubated at room temperature for 30 min. To remove detergent, Bio-Beads^®^ SM-2 Adsorbent (Bio-Rad, 400 mg) was added and incubated for 3 h with mixing at room temperature. Finally, the solution was separated from the Bio-Beads^®^ and used for Δψ measurements. To increase signal-to-noise ratio, proteoliposomes for optical experiments were prepared using the same procedure but at lipid/protein ratio of 10:1. Sodium contamination in Na^+^-free media were measured using flame photometry.

### Photoelectric responses of NaR-containing liposomes

Photoelectric responses of proteoliposomes with NaR were measured electrometrically using a phospholipid-impregnated collodion film as described previously^35^,^36^. The film separated two electrolyte-containing compartments of a Teflon chamber. This method allows the direct measurement of undistorted electric signals with ~200 ns time resolution. Proteoliposome suspension was added into one of the compartments (final concentration of NaR in the cuvette, ~7 μg/mL). To induce adsorption of proteoliposomes on the film surface, 20 mM CaCl_2_ was added. Ag/AgCl electrodes were used to measure the transmembrane electric potential difference across the membrane of adsorbed proteoliposomes. The voltage output was coupled via an operational amplifier (Burr Brown 3554BM, USA) to a CS8012 Gage and then to a computer. Light-dependent reactions in this system were induced by laser flashes (frequency-doubled YAG, 532 nm; pulse half-width, 15 ns; pulse energy, 20 mJ; Quantel, Les Ulis, France). Using this system, we can measure electric events accompanying synchronous single-turnover photoexcitation of NaR reconstituted into liposomes.

### Time-resolved spectrophotometric measurements of the NaR photocycle

Time-resolved multi-wavelength absorption changes of NaR were followed on different time scales by two home-constructed detector systems^37^,^38^. One is a CCD (charge-coupled device)-based instrument that allows recording absorption changes with time resolution of 1–16 μs between individual 433-nm-wide spectra, maximum of recording time, 2 ms. The second is a CMOS (complementary metal–oxide–semiconductor)-based detector system capable of taking 500-nm-wide spectra with pseudologarithmic time averaging starting from 14 μs per spectrum up to the completion of the photocycle. The latter detector system was built on the basis of a Sprint spL2048-140 km (Basler vision technologies) linear scan camera coupled to a CP-140–104 imaging spectrograph (Horiba Jobin Yvon). The two detector systems can be coupled to the same sample compartment with an optical fiber. Switching the optical fiber between the two detector systems allowed us to record micro- and millisecond optical changes from the same sample. The sample contained NaR (0.6 mg/mL) in Buffer A supplemented with a detergent (0.05% *n*-dodecyl β-d-maltoside) or NaR-proteoliposomes in the Buffer A. Flash photolysis was initiated by a laser flash (frequency-doubled YAG, 532 nm; pulse energy, 100 mJ; Brilliant B; Quantel, Les Ulis, France).

### Data Analysis

Basic data matrix manipulations and analyses were done with Matlab (The MathWorks, Inc.).

The curves obtained at measurements of the photoelectric responses of NaR-containing liposomes were deconvoluted by fitting employing a least square minimization procedure. The curves were described as *m* consecutive irreversible steps and fitted by Equations 2 and 3^39^,


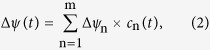



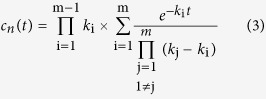


where Δψ(*t*) is the electric potential at time *t*, *m* is the number of consecutive reactions, Δψ_n_ is amplitude of the electric potential generation at the *n*^th^ reaction step, *c*_n_(*t*) is the concentration of the corresponding intermediate at time *t* (total NaR concentration was taken as 1), *k*_i_ and *k*_j_ are the rate constants for the *i*^th^ and *j*^th^ steps. These equations were derived assuming that the photocycle could be represented as a sequence of irreversible first-order reactions. The outputs of the fitting procedure included the values of Δψ_n_ (i.e., contribution of each step to the total process of Δψ generation) and the values of *k* for *m* reaction steps. The initial estimates of these parameters required to fit Equations 2 and 3 were obtained from a preliminary fit to a set of *m* independent exponential processes.

The data surfaces obtained at time-resolved spectrophotometric measurements of the NaR photocycle were deconvoluted by global fitting employing a mean absolute residual minimization procedure. The data surfaces were described as *m* consecutive irreversible steps and fitted by Equations 4 and 5^39^,^40^,


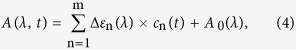



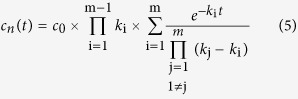


where *A*(*λ*, *t*) is the absorbance at wavelength *λ* and time *t*, *m* is the number of consecutive reactions, Δ*ε*_n_(*λ*) is the change in the extinction coefficient at wavelength *λ* in the *n*^th^ reaction step, *c*_n_(*t*) is the concentration of the corresponding intermediate at time *t, c*_0_ is total NaR concentration, *k*_i_ and *k*_j_ are the rate constants for the *i*^th^ and *j*^th^ steps, and *A*_0_(*λ*) is the final absorbance value at wavelength *λ* toward which the entire system is decaying. These equations were derived assuming that the kinetics at all wavelengths can be described by the same set of consecutive reactions. We also assumed that the photocycle could be represented as a sequence of irreversible first-order reactions. The outputs of the fitting procedure included the values of Δ*ε*_n_ at all measured wavelengths (i.e., the difference spectra) for photocycle intermediates and the values of *k* for *m* reaction steps. The initial estimates of these parameters required to fit Equations 4 and 5 were obtained from a preliminary fit to a set of *m* independent exponential processes (the SPLMOD algorithm^41^).

## Additional Information

**How to cite this article**: Bogachev, A. V. *et al.* Real-time kinetics of electrogenic Na^+^ transport by rhodopsin from the marine flavobacterium *Dokdonia sp.* PRO95. *Sci. Rep.* **6**, 21397; doi: 10.1038/srep21397 (2016).

## Figures and Tables

**Table 1:** Characteristics of three phases of Δψ generation by NaR reconstituted into liposomes.

Phase	τ at [Na^+^] = 200 mMin H_2_O	τ at [Na^+^] = 200 mMin D_2_O	Contribution tototal Δψgeneration	Dependence on Na^+^concentration
I	25 μs	35 μs	15%	No
II	0.9 ms	0.9 ms	15%	?
III	4.5 ms	9 ms	70%	Yes
